# Experimental endolymphatic hydrops under action of a type II nitric oxide synthase inhibitor: otoacoustic emissions evaluation and electrocochleography

**DOI:** 10.1016/S1808-8694(15)30049-5

**Published:** 2015-10-19

**Authors:** Claudio Marcio Yudi Ikino, Roseli Saraiva Moreira Bittar, Karina Midori Sato, Newton Macuco Capella

**Affiliations:** a MD, PhD in Sciences - FMUSP - Assistant Otorhinolaryngologist - Hospital Universitário da UFSC.; b PhD in Medicine, Assistant Physician at the Neurotology Department - Otorhinolaryngology Clinics - HCFMUSP.; c Medical Student - UFSC.; d PhD in Medicine, Assistant Professor of the Surgery Department - UFSC.

**Keywords:** endolymphatic hydrops, nitric oxide, enzimatic inhibitors, audiometry evoked response, guinea pigs

## Abstract

In experimental endolymphatic hydrops distortion-products otoacoustic emission (dpoae) amplitudes decrease and there is elevation on electrocochleographic thresholds. Some authors found type ii nitric oxide synthase (nos ii) expression in hydropic cochleas and they suggest nitric oxide (no) may be involved in endolymphatic hydrops pathogenesis. The aim of this study was to evaluate the action of a nos ii inhibitor on dpoae and electrocochleography in experimental endolymphatic hydrops. **Material and methods:** endolymphatic hydrops was induced in 16 guinea pigs by obliterating the endolymphatic duct and sac in the right ear. They were divided in two groups: eigth guinea pigs under the action of aminoguanidine, a nos ii inhibitor and eigth control guinea pigs. We compared dpoae amplitudes at geometric means of frequencies 1062, 2187, 4375 and 7000 hz, compound action potential threshold at 1000, 2000, 4000 and 6000 hz and summating potential to action potential (sp/ap) ratio between the groups during the postoperative observation period of 16 weeks. **Results:** there were no significant changes in the dpoae amplitudes and in the sp/ap ratio. The group that received aminoguanidine had a lower degree of threshold increase at 2000 (p<0.05) And 6000 hz (p<0.05) In 12th postoperative week and at 1000 (p<0.05), 2000 (P<0.001), 4000 (P<0.001) And 6000 hz (p<0.001) At 16th postoperative week. **Conclusions:** nos ii inhibitor decreased the electrocochleography threshold elevation on experimental endolymphatic hydrops.

## INTRODUCTION

The Discovery of molecular and biochemical mechanisms involved in the cochlear physiology has revolutionized concepts and opened new frontiers for research on this complex and fascinating theme. Within this context we have nitric oxide (NO), participant in a number of physiological and physiopathological processes in various systems, in the inner ear it acts in neurotransmission and bears a regulating action on the vascular tonus and on homeostasis and endolymph^1^, ^2^.

NO is synthesized from L-arginine by a family of cytoplasm enzymes called nitric oxide synthetases (NOS). These enzymes have three distinct isophorms: type I, or neuronal (NOS I), type II or induced (NOS II), and type III or endothelia (NOS III). NOS I and III are constitutional, calcium-dependent and produce small amounts of NO^3^. NOS II has its production induced by different stimulus such as cytokines, ischemia, interferon or lipopolysaccharide, it is calcium-dependent, it is not usually expressed in the cochlea and produces NO in great amounts, about 100 to 1000 times more that NOS I and III^2^, ^4^, ^5^, ^6^, ^7^.

When produced in greater amounts and continuously, NO has neurotoxic and citotoxic effects, moreover, the former is catalyzed by NOS II^2^, ^7^, ^8^, ^9^, ^10^. Studies in which NO donors are used, in other words, exogenous substances that release NO when metabolized, show that NO causes cell injury and loss of hair cells. Besides structural lesions, there are alterations in electrophysiological thresholds, endocochlear potential, auditory nerve action potential amplitude and cochlear microphone amplitude^2^, ^11^.

In a research carried out in guinea pigs with experimental endolymphatic hydrops secondary to endolymphatic sac and duct block, NOS II was detected in ganglionic cells, support cells, spiral ligament and stria vascularis. From then, it was thought that the cells were damaged by an increase in hydrostatic pressure that would release pro-inflammatory cytokines, thus stimulating NOS II synthesis and the excessive production of NO. 8 NO-related morphological and electrophysiological alterations that occur in the cochlear present similarities with the cochlear alterations that appear in animal models with endolymphatic hydrops, described in previous studies, thus suggesting a possible relationship between them^12^.

As to hearing outcomes in experimental endolymphatic hydrops, we observed a progressive deterioration of hearing levels, initially in the lower frequencies, followed by the high frequencies, and finally in the intermediate frequencies, very similar to the clinical outcome of Menière’s disease^13^, ^14^, ^15^. There is reduction in the amplitude of the distortion product otoacoustic emissions (DPOAE) which happen early on in the first weeks after the experimental induction of endolymphatic hydrops, even before the increase in thresholds detected by the compound hearing potential^16^, ^17^, ^18^.

Thus, in the endolymphatic hydrops experimental model in guinea pigs, there is cochlear lesion, progressive increase in electrophysiological threshold and drop in DPOAE amplitude, such alterations are interconnected. In this same animal model NOS II expression was found in the cochlea, and such isophorm is not normally found in the inner ear. Therefore, it is possible that the NO synthesized under the action of the NOSII may play a role in the origin of cochlear histopathological alterations, of compound auditory potential and DPOAE observed in these experimental animals and the use of a NOS II inhibitor could potentially modify the outcome of such alterations.

The goal of the present study was to assess the action of a type II Nitric Oxide Synthase, aminoguanidine, in distortion product otoacoustic emissions and electrocochleography in experimental animals with experimental endolymphatic hydrops by obliteration of the endolymphatic sac and duct.

## MATERIALS AND METHODS

This research project followed the ethical principles upheld by the Brazilian College of Animal Experimentation, and was approved by the Ethics Committee on the use of animals of the UFSC under protocol numbers 261/CEUA and 23080.035333/2003-63/UFSC and by the Ethics Committee for the Analysis of Research Projects of the University Hospital of the Medical School of the University of São Paulo (FMUSP) under protocol number 588/04.

This experimental, case-controlled study was carried out in 16 albino animals (Cavia porcellus), of short hair, adult, from both genders, with initial weight of 350 to 400g, with a positive Preyer test and normal otoscopy. All animals underwent surgery to induce experimental endolymphatic hydrops in the right ear, with endolymphatic sac and duct obliteration through an extradural approach via posterior fossa19, under intramuscular given anesthesia using a Xylazine (5 mg/kg) and ketamine (50 mg/kg)20 solution.

The animals were randomly divided in two groups:

a) Group I (Study): comprises 8 animals that received selective NOS II inhibitor, 98.5% oral aminoguanidine bicarbonate (Sigma-Aldrich, Steinheim, Germany), in the drinking water, diluted in a 1% concentration, in a volume that matched the 100 mg dose of aminoguanidine perkg of body weight per day21, daily, from the 3rd day to the 16th week of postoperative ad libitum.
b) Group II (Control): comprises 8 animals that received only water from the drinking fountain.

DPOAE and electrocochleography tests were carried out on the right ears of 16 experimental animals under anesthesia in the preop and in the 1st, 4th, 8th, 12th and 16th week of postop. Each animal underwent DPOAE measures followed by electrocochleography, always on the right ear. The animals were randomly selected for the exams and the examiner did not know to which group the animal belonged when he did the electrocochleography.

The measures of DPOAE amplitudes were carried out in sound treated rooms, using the WelchAllyn GSI 60 DPOAE device with 70DbSPL intensity and primary frequency relations 1 (f1) and 2 (f2) of 1.2 in the following frequency geometric averages: 1062, 2187, 4375 and 7000Hz. At the end of the experiment we calculated both the average and standard deviation of the distortion product amplitudes for each frequency geometric average, for each moment assessed and for each group.

The electrocochleography was carried out in a sound treated room, using an Amplaid MK 22 device, with the active transtympanic electrode in the right ear, subcutaneous reference electrode in the median occipital region and subcutaneous mass electrode in the median frontal region. The acoustic stimuli were provided by a shell-type headphone model TDH 498 placed at 5 cm from the right ear. The electrophysiological action potential was measured in the frequencies of 1000, 2000, 4000 and 6000Hz with a logon type of stimulus, of alternate polarity starting at 120 dB SPL with 10 dBSPL decrements and the SP/AP ratio with the click-type stimulus at 120 dBSPL of alternate polarity. At the end of the experiment we calculated the average and the standard deviation of the electrophysiological thresholds for each frequency, for each moment assessed and for each group. We also calculated the average and standard deviation of the SP/AP (summation potential/action potential) ratio for each moment for both groups.

In order to assess the results between the study group and the control group and the evolution along the period studied, we used the Double Factor Variance Analysis for repeated measures, complemented by the Bonferroni t test. The software used was GraphPad Prism 4 for Windows, version 4.02. We adopted a significance level (p) of 5% (p<0.05), according to the standards used in biological studies.

## RESULTS

**Figure 1:**
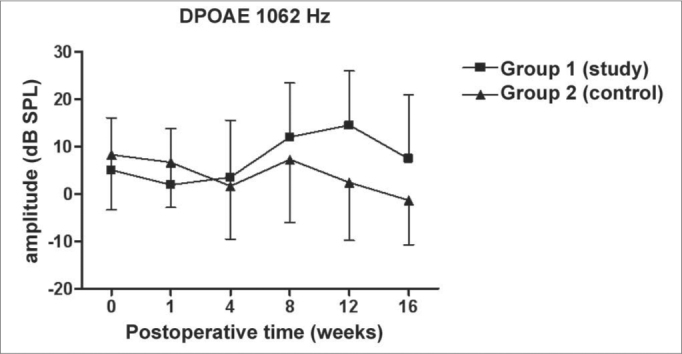
DPOAE: average of the amplitude measures in the study and control groups ± the standard deviation in the geometric averages in the frequency of 1062Hz.

**Figure 2:**
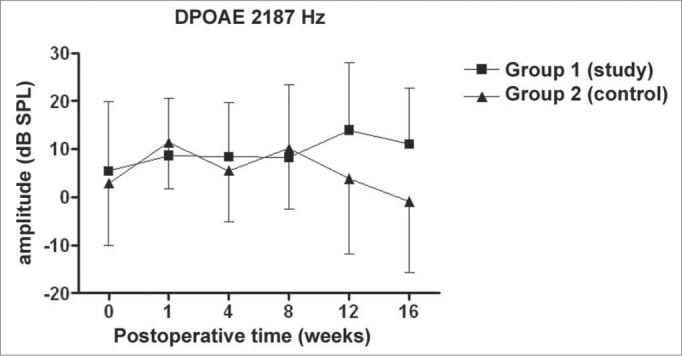
DPOAE: average of the amplitude measures in the study and control groups ± the standard deviation in the geometric averages in the frequency of 2187Hz.

**Figure 3:**
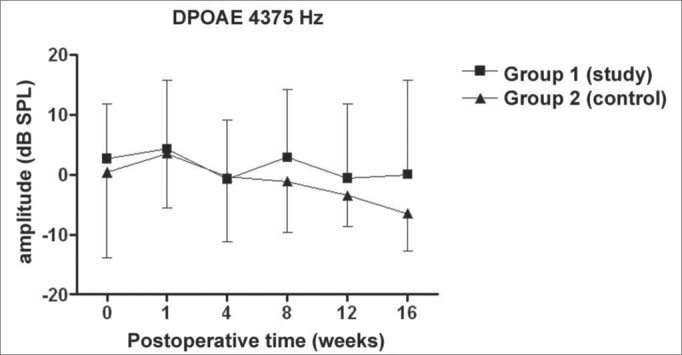
DPOAE: average of the amplitude measures in the study and control groups ± the standard deviation in the geometric averages in the frequency of 4375Hz.

**Figure 4:**
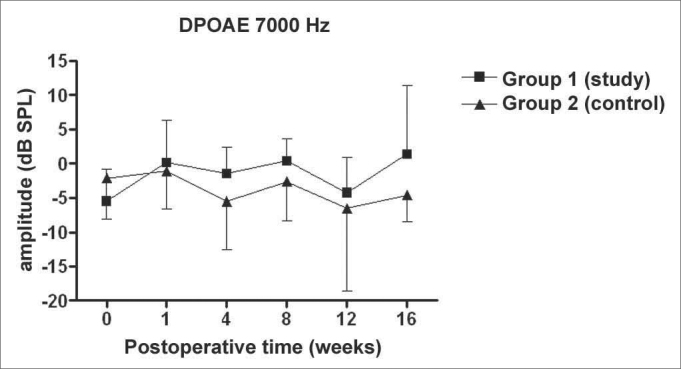
DPOAE: average of the amplitude measures in the study and control groups ± the standard deviation in the geometric averages in the frequency of 7000Hz.

We did not observe statistically significant differences in the DPOAE amplitudes in the frequencies tested along the study, nor between study and control groups (Figure 1, Figure 2, Figure 3, Figure 4).

There was a statistically significant increase in the double factor Variance Analysis of the electrophysiological thresholds seen in the electrocochleography in the following frequencies: 1000Hz (p<0.001; F=19.37). 2000Hz (p<0.001; F=21.21). 4000Hz (p<0.001; F=11.70) and 6000Hz (p<0.001; F=13.36) in both the control and study groups along the study period, pointing towards a hearing loss development and success in the endolymphatic hydrops induction.

**Figure 5:**
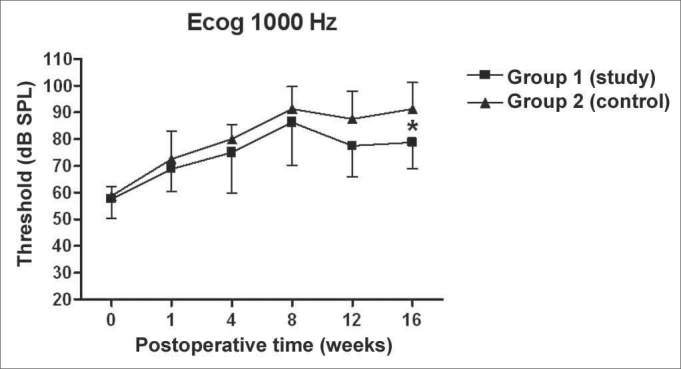
Electrocochleography: electrophysiological threshold values in average ± standard deviation in the study and control group in the frequency of 1000Hz. * p<0.05 (t=2.520) between the values of both the study and control group in the t test of Bonferroni.

**Figure 6:**
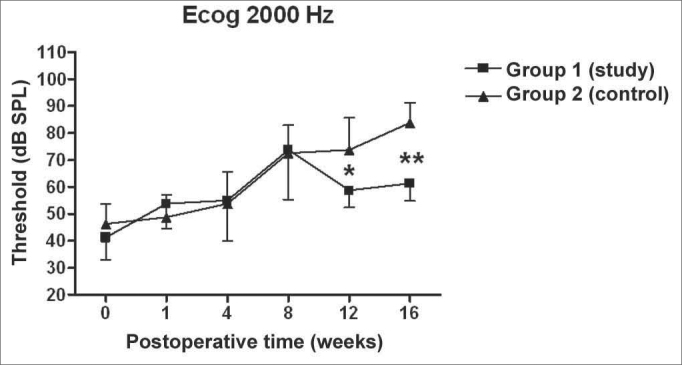
Electrocochleography: electrophysiological threshold values in average ± standard deviation in the study and control group in the frequency of 2000Hz. * p<0.05 (t=4.535) between the values of both the study and control group in the t test of Bonferroni.

**Figure 7:**
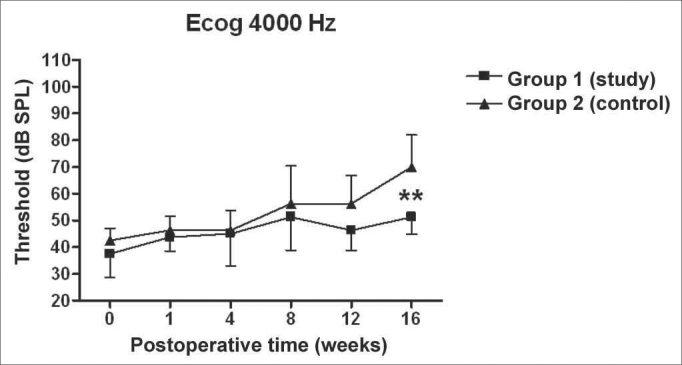
Electrocochleography: electrophysiological threshold values in average ± standard deviation in the study and control group in the frequency of 4000Hz. * p<0.001 (t=3.779) between the values of both the study and control group in the t test of Bonferroni.

**Figure 8:**
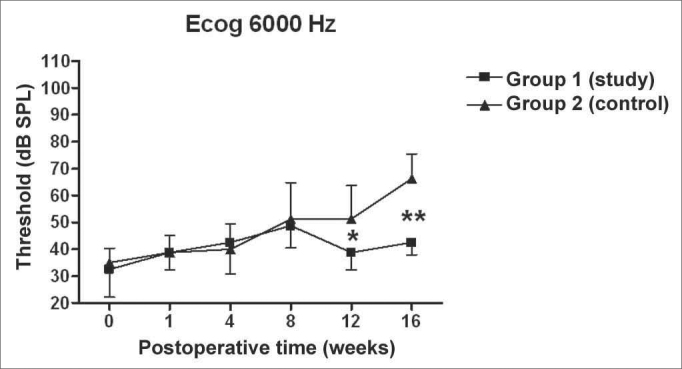
Electrocochleography: electrophysiological threshold values in average ± standard deviation in the study and control group in the frequency of 6000Hz. * p<0.05 (t=2.520); p<0.001 (t=4.787) between the values of both the study and control group in the t test of Bonferroni.

**Figure 9:**
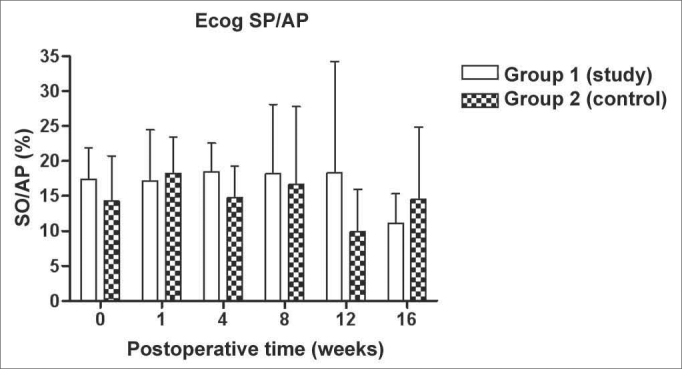
Electrocochleography measured from the SP/AP ration in average ± the standard deviation in both the study and the control group.

Figure 5, Figure 6, Figure 7, Figure 8 depict the measures of electrophysiological thresholds, and compare the values between control and study group along the study period. Figure 9 shows the SP/AP ratio behavior in the study and control group where there was no statistically significant difference among them and in their values along the study period.

## DISCUSSION

The DPOAE amplitude measures did not show variations along the study in neither the control nor the study group. The sample size, together with the great individual variability may have had an impact there. In some studies, DPOAE amplitude in the endolymphatic hydrops experimental model shows a reduction already in the 1st week of postoperative time^16^, ^18^ while others, as it happened in our experiment, showed variable results, even with an amplitude increase after the reduction period, however it did not reach preoperative values^17^, ^22^. Also, the intensity of the primary frequencies used varies according to the authors, making it difficult to compare the results. On the other hand, the amplitude alterations, when detected, precede the electrophysiological threshold alterations^16^, ^17^ thus, there may be other mechanisms responsible for its origin, and NOS II would not play an important role. Okubo et al. (1997)^18^ showed that the DPOAE amplitudes in 4000 and 6000Hz showed a reduction after 1 to 2 weeks of postoperative time, which returned to their baseline preoperative values with the use of osmotic antidiuretic agent, isosorbide, thus suggesting the role of endolymph pressure and volume in the DPOAE alterations. In this same study the endocochlear potential also reduced in the first week of postop, however isosorbide brought about a greater reduction in its measures, not showing any apparent relation to DPOAE^18^.

We have seen a progressive increase in electrophysiological thresholds in the control group in all the frequencies along the study period, initially in 1000 Hz already in the first week of postop, and in the remaining frequencies it started on the 8th week. Our findings are similar to those from other authors, confirming how adequate this animal is as an experimental model for endolymphatic hydrops^13^, ^16^, ^23^, ^24^, ^25^. Thresholds were higher than preoperative measures, probably because we provided the acoustic stimuli through a head phone placed at 5cm from the ear while others used it at 1cm^22^, ^24^, ^26^ or fit the phone to the external auditory canal^13^. Even then, the hearing loss pattern and level seen were similar. As to preoperative values, our results agree with those from Andrews et al. (2000)^26^. We tried to place the earphone as close as possible to the ear; however, the size of the transtympanic electrode used did not let us go closer.

In the study group there was an increase in electrophysiological thresholds in the frequencies of 1000 and 2000Hz in the first 4 weeks of postop, reaching the remaining frequencies on the 8th week, in a similar pattern to that of the control group. However, starting on the 12th week the hearing loss progression was less in the study group, being significant in 2000 and 6000 Hz, including the measures in 1000 and 4000 Hz on the 16th week. Therefore, oral aminoguanidine was able to reduce the hearing loss caused by endolymphatic hydrops in experimental animals as of the 12th week postoperative, but did not preclude its development.

Aminoguanidine is a selective NOS II inhibitor^27^. Its intraperitoneal use in the dose of 100 mg/kg/day reduced tissue lesions and the increase in electrophysiological thresholds in experimental animals that had their cochleas submitted to transitory ischemia by clamping the labyrinthine artery, thus proving that aminoguanidine does reach the cochlear tissue^21^. Studies utilizing oral aminoguanidine bicarbonate in doses varying from 80 to 200 mg/kg/day, showed that the drug is absorbed by the GI tract and acts inhibiting NOS II action, because it reduces the lesions produced in disease models in which its expression increase had been proved, and also reducing the urinary excretion of nitrites and nitrates, pointing towards a lower NO production. We believe that in our study aminoguanidine inhibited NOS II and, as we observed a reduction in the electrophysiological thresholds, we suggest that NO catalyzed by this enzyme plays a role in the pathogenesis of endolymphatic hydrops. Michel et al. (2000)^8^ and Watanabe et al. (2001)^10^ showed that there is NOS II expression in the cochlea of experimental animals with hydrops, suggesting its participation in disease development. When an inhibitor of this enzyme is used we were able to change the hearing loss development, and this has come to reinforce that assumption. Notwithstanding, in order to compare, we did not find in the literature the use of NOS II inhibitor, either per os or parenteral in any endolymphatic hydrops experimental model.

Although we did see a lower level of hearing loss in the study group, there was an increase in electrophysiological thresholds, therefore, aminoguanidine did not prevent endolymphatic hydrops development. The fact that we started aminoguanidine on the 3rd day of postop may have caused it, because then we allowed NOS II to produce NO in the first two days. Our decision for starting aminoguanidine as of that date was due to a risk of local infection, because the surgery used to cause endolymphatic hydrops involved the dura mater, the occipital bone and the internal ear. Since NOS II participates in the inespecific cell mechanisms for defense and inflammation^4^, we decided to wait 2 days, thus allowing healing of the surgical wound. We do not know for sure when NOS II is synthesized, since Michel et al. (2000)^8^ showed this enzyme present in experimental animals at 3 weeks of the hydrops induction by a block in both the endolymphatic duct and sac, while Watanabe et al. (2001)^10^ showed NOS II expression already at 1 day of postop. In this last model, the authors used antigen injection in the endolymphatic sac to cause hydrops and we do not know if NOS II expression occurs at the same time in these two different induction models. In guinea pigs in which we produce cochlear lesions, either by LPS or by gentamicin, NOS II is responsible for NO synthesis 48 hours after inoculating these toxic agents^29^. Another possibility is the use of oral aminoguanidine in the maximum dose of 100 mg/kg/day that may not have reached enough concentration for total NOS II inhibition. Oral use proved to be effective in other disease models in doses varying between 80 and 200 mg/kg/day and in the dose of 100 mg/kg/day - that we used, in experimental animals exposed to cochlear ischemia, however via intraperitonium^21^, ^28^. We are not certain whether this dosage was enough in endolymphatic hydrops, since we did not find other experiments that used it in this disease model. Our method did no include drug ingestion control that may have varied, as well as its serum level maintenance; notwithstanding, we observed that most animals, during the study period, drank all or almost all the drinking fountain water. And finally, there is the possibility that endolymphatic hydrops be of multifactorial pathogeny. Thus, aminoguanidine would not prevent hydrops onset, as we saw in our experiment in a significant way as of the 12th week. The mechanical obstruction of the endolymphatic sac and duct^19^, ^30^ in experimental animals promotes endolymphatic hydrops with endolymphatic space distention already on the 1st day. In this model there is an increase in endolymph pressure in comparison to the perilymph after the 2nd week 26 which may cause hearing loss induced by the mechanical action or by cell lesion. The use of diuretics, such as glycerol^25^, is capable of reducing electrophysiological thresholds increase, but it also does not prevent the disease onset, suggesting that the increase in endolymphatic pressure does play a role, but it is not the only factor involved. In order to reinforce this aspect, studies that promote the increase in endolymphatic pressure through a catheter positioned within the endolymphatic duct, assessing the endolymphatic pressure factor alone caused hearing loss, initially on the high frequencies, and later on reaching the lower frequencies. Now, in the hydrops caused by endolymphatic duct and sac block, the lower frequencies hearing loss is the first to occurr^24^. These aforementioned alterations may be responsible or may happen together with NOS II expression, with NO production damaging hair cells and also the marginal cells of the stria vascularis and over activate the ON/GMPc via with consequent cochlear microcirculation dysfunction and greater glutamate release. The latter would block neural transmission through N-metil-D-aspartato receptors^2^, ^7^, ^8^, ^10^, ^29^. Aminoguanidine was probably capable of mitigating these lesions.

We did not find any difference as far as the SP/AP ration is concerned in the control group, like other authors^14^, ^23^ who also used the active lead in the base of the cochlea. Aminoguanidine did not change this finding. The mechanisms responsible for hearing alterations in experimental endolymphatic hydrops are various and not yet fully understood. NO comes up as one more element involved in the disease genesis, and new studies to clarify its role, as well the interaction with the other factors presented in the literature are necessary. A better understanding of the endolymphatic hydrops physiopathology is the basis to develop new therapeutic approaches for the Menière Disease.

## CONCLUSIONS

We have concluded that aminoguanidine, NOS II inhibitor, caused a milder increase in the electrophysiology thresholds of experimental animals, and did not change the SP/AP ratio measured at the base of the cochlea; and also did not change DPOAE amplitudes along the 16 weeks of the study.
